# Genomic and transcriptomic comparison of nucleotide variations for insights into bruchid resistance of mungbean (*Vigna radiata* [L.] R. Wilczek)

**DOI:** 10.1186/s12870-016-0736-1

**Published:** 2016-02-17

**Authors:** Mao-Sen Liu, Tony Chien-Yen Kuo, Chia-Yun Ko, Dung-Chi Wu, Kuan-Yi Li, Wu-Jui Lin, Ching-Ping Lin, Yen-Wei Wang, Roland Schafleitner, Hsiao-Feng Lo, Chien-Yu Chen, Long-Fang O. Chen

**Affiliations:** Institute of Plant and Microbial Biology, Academia Sinica, 128 Sec. 2, Academia Rd, Nankang, Taipei 11529 Taiwan; Department of Bio-Industrial Mechatronics Engineering, National Taiwan University, Taipei, 106 Taiwan; AVRDC-the World Vegetable Center, 60 Yi-min Liao, Shanhua, Tainan, 74151 Taiwan; Department of Horticulture and Landscape Architecture, National Taiwan University, Taipei, 106 Taiwan

**Keywords:** *Vigna radiata*, *Callosobruchus* spp., Next generation sequencing, Differential expressed gene, Nucleotide variation, Molecular marker

## Abstract

**Background:**

Mungbean (*Vigna radiata* [L.] R. Wilczek) is an important legume crop with high nutritional value in South and Southeast Asia. The crop plant is susceptible to a storage pest caused by bruchids (*Callosobruchus* spp.). Some wild and cultivated mungbean accessions show resistance to bruchids. Genomic and transcriptomic comparison of bruchid-resistant and -susceptible mungbean could reveal bruchid-resistant genes (*Br*) for this pest and give insights into the bruchid resistance of mungbean.

**Results:**

Flow cytometry showed that the genome size varied by 61 Mb (mega base pairs) among the tested mungbean accessions. Next generation sequencing followed by *de novo* assembly of the genome of the bruchid-resistant recombinant inbred line 59 (RIL59) revealed more than 42,000 genes. Transcriptomic comparison of bruchid-resistant and -susceptible parental lines and their offspring identified 91 differentially expressed genes (DEGs) classified into 17 major and 74 minor bruchid-resistance–associated genes. We found 408 nucleotide variations (NVs) between bruchid-resistant and -susceptible lines in regions spanning 2 kb (kilo base pairs) of the promoters of 68 DEGs. Furthermore, 282 NVs were identified on exons of 148 sequence-changed-protein genes (SCPs). DEGs and SCPs comprised genes involved in resistant-related, transposable elements (TEs) and conserved metabolic pathways. A large number of these genes were mapped to a region on chromosome 5. Molecular markers designed for variants of putative bruchid-resistance–associated genes were highly diagnostic for the bruchid-resistant genotype.

**Conclusions:**

In addition to identifying bruchid-resistance-associated genes, we found that conserved metabolism and TEs may be modifier factors for bruchid resistance of mungbean. The genome sequence of a bruchid-resistant inbred line, candidate genes and sequence variations in promoter regions and exons putatively conditioning resistance as well as markers detecting these variants could be used for development of bruchid-resistant mungbean varieties.

**Electronic supplementary material:**

The online version of this article (doi:10.1186/s12870-016-0736-1) contains supplementary material, which is available to authorized users.

## Background

Mungbean (*Vigna radiata* [L.] R. Wilczek) is an important legume crop with high nutritional value in South and Southeast Asia. Because of its high content of easily digestible protein and relatively high iron and folate contents, it represents a nutrition-balanced food for cereal-based diets [1–3]. Mungbean is also consumed as sprouts, which are important sources of vitamins and minerals [4, 5].

Bruchids (*Callosobruchus* spp.), the bean weevils, cause serious damage to and loss of legume seeds, including mungbean, during storage [6]. Infestation of the crop is generally low in the field. Only a few insect-infested seeds are needed for the initial inoculum for population build-up during grain storage [7]. Bruchid development from eggs to pupae takes place in a single seed, the larva being the most destructive stage. The emerging adults deposit eggs on the seed, causing rapid multiplication of the pest during storage and resulting in up to 100 % of grain loss.

Only a few bruchid-resistant mungbean varieties are available today [8] and resistant lines adapted to the tropics are lacking. Chemical controls with organophosphate compounds, synthetic pyrethroids or insect growth regulators widely used to protect mungbean against this pest [9] are expensive, with risks to consumer health and the environment and development of insecticide tolerance by the pest [10, 11]. Biological control by the bruchid parasitoid *Dinarmus* sp. is less efficient than chemicals in reducing the storage pest effects [12]. Therefore, host resistance would be the most sustainable and economical way to preserve mungbean seeds against destruction by bruchids during storage.

The wild mungbean accession *V. radiata* var. *sublobata* TC1966 from Madagascar is resistant to many bean weevil species, including *Callosobruchus chinensis*, *C. phaseoli*, *C. maculatus*, and *Zabrotes subfasciatus* [13, 14]. TC1966 is easily crossed with *V. radiata*, and the bruchid resistance of this accession was introduced into the cultivated gene pool [14–16]. The bruchid resistance of TC1966 was proposed to depend on a single dominant gene plus one or a few modifier factors [16–19]. A bruchid-resistant gene (*Br*) for this line has not yet been identified, although several candidate genes have been suggested and genetic markers co-segregating with the *Br* gene have been described. On restriction fragment length polymorphism (RFLP) analysis of 58 F2 progenies from a cross of TC1966 and a susceptible mungbean, VC3890, the *Br* was mapped to a single locus on linkage group VIII, approximately 3.6 centimorgans (cM) from the nearest RFLP marker [19]. Later, 10 randomly amplified polymorphic DNA (RAPD) markers were found associated with the *Br* gene in a segregating population derived from TC1966 and NM92, a bruchid-susceptible mungbean [15]. RNA-directed DNA polymerase, gypsy/Ty-3 retroelement and chloroplast NADH dehydrogenase subunit genes were highly associated with the proposed *Br* gene of mungbean [15]. Further quantitative trait loci (QTL) analysis revealed one major and two minor QTL for bruchid resistance in TC1966 [17]. Seed metabolite analysis in line BC20F4 derived from a cross between TC1966 and a susceptible cultivar Osaka-ryokuto suggested the involvement of cyclopeptide alkaloids named vignatic acids with bruchid resistance. The gene responsible for vignatic acid (*Va*) accumulation was mapped to a single locus, 0.2 cM away from the previously mapped *Br* gene [20]. Additionally, a small cysteine-rich protein, VrCPR protein, which is lethal to *C. chinensis* larvae, was identified in TC1966 [21]. Proteomic research has proposed that chitinase, beta-1,3-glucanase, peroxidase, provicilin and canavalin precursors play a role in bruchid resistance of mungbean [22].

The implication of proteinase and amylase inhibitor activity in bruchid resistance in legumes remains controversial [23–26]. Whether these candidate factors indeed associated with previously described bruchid-resistant QTL [17] and contributed to resistance remained unknown. Some of the putative *Br* factors of TC1966 may be harmful for human consumption [27]. Because the chemical nature of the resistance factor is still unknown, the safety of using the resistance factors derived from TC1966 is difficult to assess. Despite much effort directed toward the identification of bruchid-resistant factors, physiological differences between bruchid-resistant and -susceptible mungbean have not been reported.

A molecular marker associated with *Br* would facilitate breeding of bruchid-resistant varieties, and mapping of the resistance genes also would help identify factors underlying resistance. Available markers have not been validated for breeding, and more information on *Br* is required to generate reliable markers for breeding bruchid-resistant mungbean varieties. Gene-based or regulatory sequence-based markers would be the most efficient for selecting bruchid-resistant lines in breeding programs. In contrast to resistance locus-linked RFLP and RAPD markers, resistance-gene or regulatory sequence-based markers cannot be separated from the resistant phenotypes by recombination and thus are more reliable for selection. Bruchid resistance is assumed to be due to the expression of resistance factors. Resistance factors could be direct products of resistance genes that are absent in susceptible lines, or could result from activity changes of factors in susceptible and resistant lines due to sequence variation or from expression differences of resistance genes. Polymorphisms related to any of these differences would provide reliable markers for resistance.

Recently, the whole-genome sequence of a bruchid-susceptible mungbean (*V. radiata* var. *radiata* VC1973A) was published [28]. Here we report the whole-genome sequence of a bruchid-resistant recombinant inbred line (RIL) and an increased number of available gene annotations for mungbean, by 14,500 genes. We have identified differentially expressed genes (DEGs) and nucleotide variations (NVs) in the promotor regions of DEGs and in the exons of sequence-changed protein genes (SCPs). The putative effects of DEGs and SCPs on bruchid resistance of mungbean are discussed and molecular markers derived from NVs that can be used for selection of resistant lines are reported.

## Results

### Genome size of different mungbean cultivars and wild relatives

The genome size estimated by cytometry ranged from about 494 to 555 Mb (mega base pairs) (Table 1) in the lines under investigation. We found about a 20-Mb difference in genome size between wild mungbean TC1966 (494 Mb) and the cultivar NM92 (517 Mb). The genome size of RIL59, offspring of a cross between TC1966 and NM92, was similar to that of its female parent NM92, whereas k-mer frequency distribution analysis of RIL59 suggested a genome size of 452 Mb. The estimated genome size of the buchid-susceptible mungbean line VC1973A, recently sequenced [28], was about 502 Mb, similar to the size of the bruchid-resistant mungbean line V2802; another bruchid-resistant mungbean line, V2709, had the largest genome size in our study.

**Table 1:** Genome size of mungbean varieties

	TC1966	NM92	RIL59	V1973A	V2709	V2802
pg/2C^a^	1.01 ± 0.02	1.06 ± 0.02	1.06 ± 0.00	1.03 ± 0.02	1.13 ± 0.01	1.04 ± 0.01
Genome size (Mb)	493.6 ± 3.3	517.3 ± 3.1	517.1 ± 0.9	502.2 ± 2.9	554.7 ± 2.8	506.1 ± 2.3

Data are mean ± SE from six biological repeats

^a^DNA content of diploid organisms (2C) represented in picograms (pg); 1 pg = 978 Mb [33]

### *De novo* genome assembly of RIL59

The previously published whole genome sequence for mungbean is derived from the bruchid-susceptible cultivar VC1973A [28]. For genomic comparison and to facilitate research on bruchid resistance of mungbean, we sequenced and assembled the draft genome of the bruchid-resistant line RIL59, whose *Br* gene was inherited from the wild mungbean accession TC1966. Sequencing of four DNA libraries, including two paired-end and two mate-pair libraries with various fragment lengths (Additional file 1: Table S1), resulted in 90.1 Gb (Giga base pairs) of sequence information, which corresponds approximately to a 174.2-fold sequencing coverage according to the genome size of RIL59 (Table 1). *De novo* assembly of the sequence reads resulted in 2509 scaffolds with an N50 of 676.7 kb (kilo base pairs) comprising 455.2 Mb (Table 2) and contributed to approximately 88 % of the estimated genome size of RIL59 (Table 1). The largest scaffold had a length of 4.4 Mb.

**Table 2:** Summary of *de novo* genome assembly of RIL59

Stage	N50 (kb)	Average Length (kb)	Total Length (Mb)	Longest (kb)	No. sequences
Contigs	34.9	16.9	437.9	287.0	25,895
Scaffolds	676.7	181.4	455.2	4419.0	2509

### Gene annotation

In total, 40.5 % of the draft genome was classified as repeat sequences and 23.3 % as long tandem repeat (LTR) elements. The repeat elements were annotated by using the TIGR plant repeat database (Table 3). Sixteen paired-end RNA libraries (Additional file 1: Table S1) constructed from RIL59 tissues and different RIL seeds represented 134.4 Gb. RNA-seq data for RIL59 (Additional file 1: Table S1) and NCBI (http://www.ncbi.nlm.nih.gov/) soybean refseq protein sequences were aligned to the repeat-masked genome to identify splice junctions for gene prediction. Overall, 63.35 % of the RNA-seq data mapped uniquely to splice junctions. *Ab initio* gene prediction combined with protein alignment resulted in annotations for 36,939 protein-coding genes; 4493 of these encoded for multiple isoforms, for 42,223 transcripts in total. Overall, 85 % of the 49,952 predicted-gene models had matches in the NCBI non-redundant protein database. The predicted-gene models consisted of transcript lengths of 4108 bp, coding lengths of 1290 bp, and 5.76 exons per gene, on average.

**Table 3:** Repeated sequences annotation of repeat elements from the TIGR database

Class	Number	Size (bp)
Retrotransposon	544	81,182
Transposon	145	21,405
Miniature Inverted-repeat Transposable Elements (MITE)	2	230
Centromere satellite	9	1161
Unclassified centromere sequence	7	1621
Telomere sequence	8	1358
Telomere associated	9	918
rDNA 45S	29	11,783
rDNA 5S	42	6914
Unclassified (total)	243	24,467

### Identification of bruchid-resistance–associated genes by transcriptome comparison

**Fig. 1:**
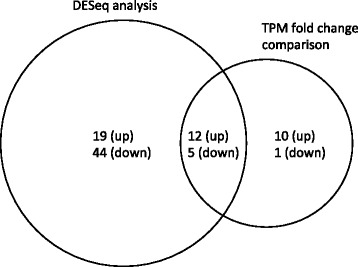
Transcriptome analysis of bruchid-resistant–associated genes in mungbean. Bruchid-resistant–associated genes were selected from transcripts per million (TPM) fold change comparison and DESeq analysis of transcriptomes between brucnid-resistant and -susceptible mungbean. The number of DEGs selected by each criterion is indicated. Up and down represent the genes up- and downregulated, respectively, in bruchid-resistant mungbean

We searched for bruchid-resistance–associated genes by comparing the seed transcriptome of bruchid-resistant (R) and -susceptible (S) mungbean lines (Additional file 1: Table S1), including two parental lines of a population of NM92 (S) and TC1966 (R), and RILs derived from this population: RIL59 (R) and three pairs of RILs, each pair with contrasting bruchid resistance. Two methods to identify DEGs by RNA-seq were applied. The first approach, which involved calculating the number of transcripts per million (TPM), revealed 22 up- and 6 downregulated genes in seeds of bruchid-resistant mungbean (Fig. 1 and Additional file 2: Table S2). Three of the upregulated genes (*g4706*, *g34480* and *g42613*) were specifically detected in R mungbean, and two downregulated genes (*g40048*, *g41876*) were specifically detected in S mungbean.

The second approach by DESeq analysis [29] of the same nine transcriptomes identified 81 transcripts of 80 DEGs; 31 were up- and 49 downregulated in bruchid-resistant mungbean (Fig. 1 and Additional file 2: Table S2). The downregulated gene *g16371* was present in two splice forms, *g16371.t1* and *g16371.t2*. Ten genes (*g24427*, *g34321*, *g4706*, *g34480*, *g28730*, *g17228*, *g9844*, *g39181*, *g39425*, *g42613*) were expressed only in bruchid-resistant lines and three (*g40048*, *g35775*, *g2158*) only in bruchid-susceptible lines. Together, the two approaches identified 91 DEGs most likely related to bruchid resistance. We classified the 17 consensus genes pinpointed by both approaches as major bruchid-resistance–associated genes and the other 74 as minor bruchid-resistance–associated genes (Fig. 1).

The 17 consensus genes are most likely highly related to bruchid resistance of mungbean, especially the 12 upregulated genes (Fig. 1 and Additional file 2: Table S2). However, five of these genes have unknown function, including three with no hits on Blastx analysis. The putative UBN2_2 domain of g34480 and RVT_2 domain of g4739 implying their transposase activity, together with the putative gag/pol polyprotein, g34458, represented transposable elements (TEs). The remaining genes encoded a putative MCM2-related protein, a putative adenylate cyclase, a senescence regulator and a resistant-specific protein (Additional file 2: Table S2).

**Fig. 2:**
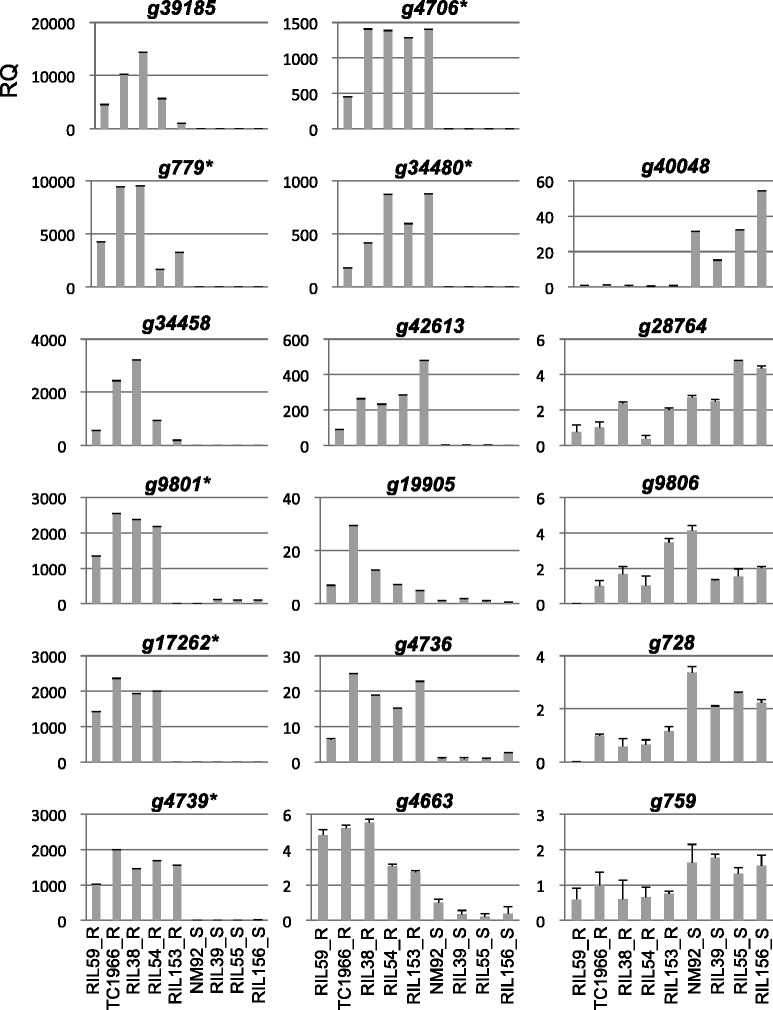
RT-qPCR validation of differentially expressed genes (DEGs). RT-qPCR results of the pattern of gene expression between bruchid-resistant and -susceptible mungbean. The Y axis indicates the relative quantity (RQ) of gene expression with mungbean *VrActin* (*g12676*) used as a control. Data are RQ ± SE of ΔΔCT from three experimental repeats. The X axis indicates different bruchid-resistant (R) and -susceptible (S) mungbean lines. Asterisk indicates that the expression of the gene was not detected in the parental line NM92 with CT value set to 40 cycles for calculating the RQ of gene expression

RT-qPCR analysis of the RNA-seq data verified the 17 consensus genes (Fig. 2). Ten upregulated genes were in all R mungbean lines as compared with S lines, except *g9801* and *g17262* were undetected in the bruchid-resistant RIL153. Among the five downregulated genes, *g40048*, *g28764* and *g759* were consistently downregulated in all R lines as compared with S lines.

**Fig. 3:**
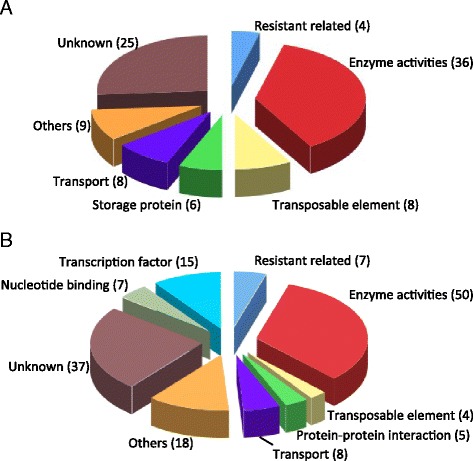
Pie chart representing the functional categories of DEGs and sequence-changed-protein genes (SCPs). DEGs (**a**) and SCPs (**b**) were functionally classified into categories based on annotation and the putative protein domains they harbored. The number of genes in each category is indicated in parentheses

The high consistency between RNA-seq and RT-qPCR results implied the DEGs might represent the biological difference between R and S mungbean seeds. In terms of functional categorization based on gene annotation combined with predicted protein domains, 36 of the 91 DEGs encoded proteins with enzymatic activities, four encoded resistant-related proteins and eight encoded TEs (Fig. 3a). Among the DEGs, 18 were involved in metabolic pathways, genetic information processing, environmental information processing and cellular processes (Additional file 3: Table S3).

Two of the DEGs, *g728* and *g17654*, encoded a cysteinyl endopeptidase and a basic 7S globulin 2 precursor, respectively. The former has protease activity and the latter was implicated in bruchid resistance [22]. Both proteins are predicted to contain an inhibitor domain (Additional file 2: Table S2). However, we found their expression downregulated in bruchid-resistant mungbean (Additional file 2: Table S2), which suggests that these proteins have no role in resistance.

NVs in promoter regions might affect the expression of genes. A survey of NVs including substitutions and insertions and deletions (indels) by comparing genomic sequences of bruchid-resistant and -susceptible lines revealed that 408 NVs located in the 2-kb region presumably included the promoter regions of 68 consensus DEGs (Additional file 4: Table S4). The number of NV sites in the 2-kb regions ranged from 1 to 24 (Additional file 4: Table S4).

### Identification of bruchid-resistance–associated SCPs

**Fig. 4:**
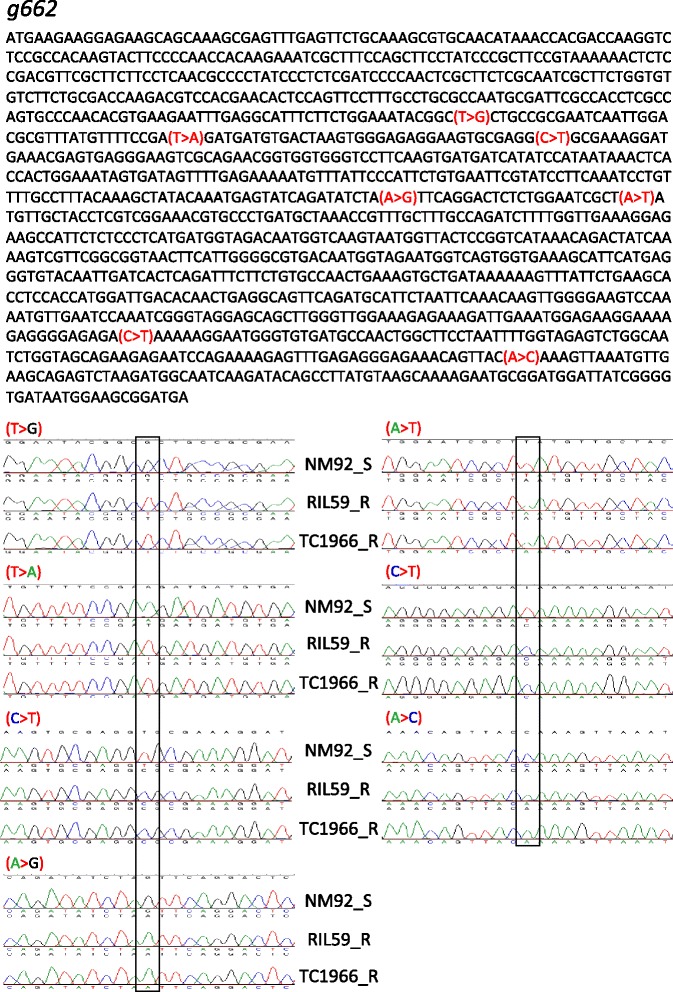
Validation of nucleotide variations (NVs) identified by RNA sequence comparison. Gene *g662* was used to illustrate the verification of NVs. The upper panel shows the cDNA sequence of *g662* and the seven NVs (mark in red) identified by RNA sequence comparison of bruchid-resistant (R) and -susceptible (S) mungbean. The NVs in parentheses show the nucleotides in R mungbean (the former letter) changed to that in S mungbean (the latter letter). The lower panel shows the validation of NVs by genomic sequencing between R mungbean lines RIL59 andTC1966 and S line NM92. The color of the letter is synchronized with that of the chromatogram for easy reading. The box indicates the site of NVs. The order of NV sites starts from down-left then down-right panels

In addition to DEGs, NVs including nonsynonymous substitutions and indels in exon regions producing SCPs can modify protein functions, without necessarily changing gene expression. Because genetic codes stored in RNA are directly transmitted to proteins, we compared NVs of genes based on RNA-seq data between bruchid-resistant and -susceptible lines and found 282 consensus NVs on 149 transcripts (148 genes) (Additional file 5: Table S5). The confidence of NVs was verified by genomic sequence comparison of a few genes between RIL59 and its parents. For illustration, seven NVs were proposed on *g662* cDNA by RNA-seq comparison (Fig. 4). Genomic sequence results confirmed that these NVs consistently exist in R mungbean, lines RIL59 and TC1966, and S mungbean, NM92 (Fig. 4).

Of the 148 SCPs, 134 could be functionally annotated by Blast analysis. Most encoded proteins harbored enzymatic activities, and 15 encoded transcription factors. Importantly, seven and four genes encoded resistant-related proteins and TEs, respectively (Fig. 3b and Additional file 6: Table S6). Similar to DEGs, 28 of the 148 SCPs were involved in pathways of metabolism, genetic information processing, environmental information processing, cellular processes and organismal systems (Additional file 7: Table S7). DEGs and SCPs involved in conserved pathways implied the conserved intrinsic difference between R and S mungbean (Additional file 3: Table S3 and Additional file 7: Table S7).

Two of the SCPs, *g29024* and *g4649*, encoded putative pectinesterase inhibitor 3-like and Kunitz trypsin inhibitor protein, respectively. Whether they are involved in bruchid resistance needs further investigation.

### Mapping of bruchid-resistance–associated NVs in the mungbean genome

Bruchid-resistance–associated DEGs and SCPs are potential *Br* genes. Hence, the NVs in the promoter region are potential regulatory-sequence–based markers, whereas NVs on SCPs are potential gene-based markers for resistance. We mapped the identified bruchid-resistance–associated NVs and genes to the mungbean genome of VC1973A [28] to assess whether their genomic position co-localizes with previously reported bruchid-resistance–associated markers. The 2-kb promoter region considered to have putative regulatory sequences implicated in resistance for the 68 DEGs was mapped to pseudochromosomes of mungbean [28]. The promoters of these DEGs were found unevenly distributed over the 11 chromosomes, and most sequences were mapped to chromosome 5 and to 10 scaffolds (Table 4 and Additional file 2: Table S2). Similarly, 282 NVs of 148 SCP genes were unevenly distributed over the 11 chromosomes and 16 scaffolds of the mungbean reference sequence [28]. Interestingly, most of these sequences were mapped to chromosome 5 (Table 4 and Additional file 6: Table S6). Therefore, 690 bruchid-resistance–associated NVs were mapped to 11 chromosomes and 21 scaffolds of the reference sequence (Table 4, Additional file 2: Table S2 and Additional file 6: Table S6).

**Table 4:** Mapping of bruchid-resistance–associated genes on mungbean pseudochromosome

	Vr1	Vr2	Vr3	Vr4	Vr5	Vr6	Vr7	Vr8	Vr9	Vr10	Vr11	Scaffolds	Total
DEGs	4	4	3	4	16	3	6	3	3	2	2	17	67
SCPs	7	3	7	10	55	6	5	1	7	1	2	44	148
Total	11	7	10	13	67	9	11	4	10	3	3	60	208

The promoter 2-kb sequences of differentially expressed genes (DEGs) and sequences of sequence-changed-protein genes (SCPs) were mapped on the 11 pseudochromosomes (Vr1 ~ Vr11) and scaffolds of mungbean [28]. The total number of genes mapped to Vr4, Vr5, Vr11 and scaffolds were not equal to the sum of DEGs and SCPs because some SCPs also belonged to DEGs

**Fig. 5:**
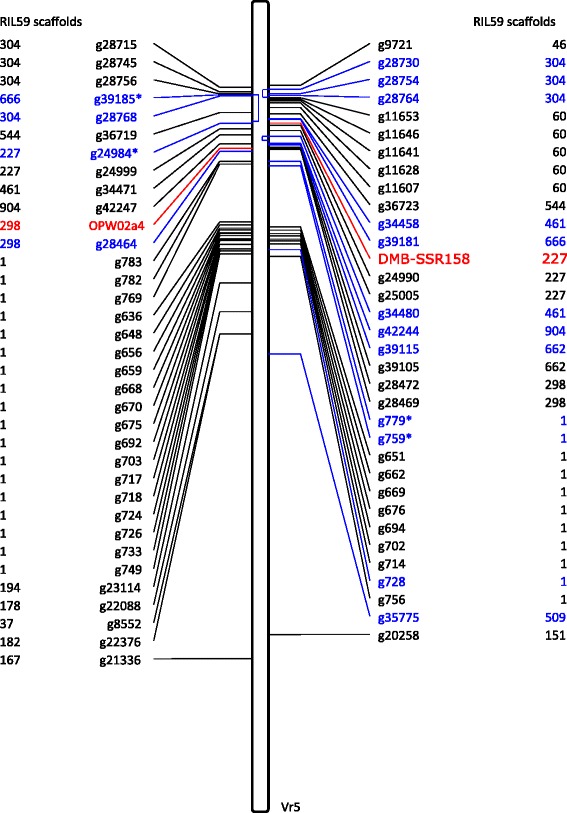
Map of bruchid-resistant–associated genes on chromosome 5 (Vr5) of VC1973A. The corresponding scaffold for each gene in RIL59 is at both sides. The two bruchid-resistant markers are in red. The DEGs are indicated in blue and SCPs in black. The DEGs with an asterisk are also SCPs. For DEGs, the 2-kb promoter sequences were used for mapping, whereas for SCPs, the gene sequences were used

**Fig. 6:**
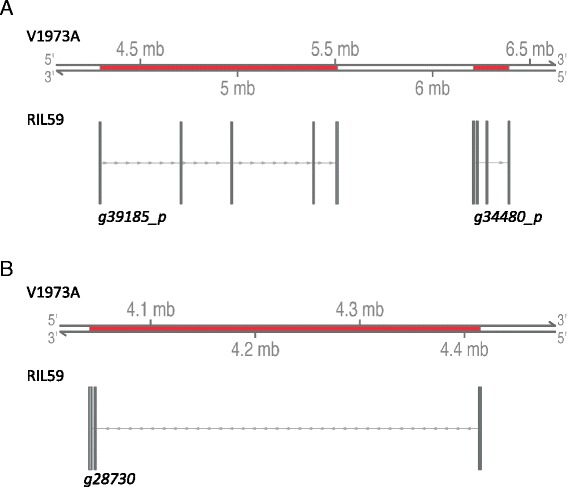
Close-up map of *g39185* and *g34480* promoter sequence (**a**) and *g28730* gene (**b**) on mungbean Vr5. The 2-kb promoter sequences of *g39185* (*g39185_p*) and *g34480* (*g34480_p*) and *g27830* gene of RIL59 are strikingly different from that of VC1973A. The number on Vr5 of V1973A indicates the position on the chromosome. mb, million base

The two published bruchid-resistance–associated markers, the cleaved amplified polymorphic DNA (CAP) marker OPW02a4 and the simple sequence repeat (SSR) marker DMB-SSR158 [15, 17], were mapped to scaffolds 298 and 227 of RIL59, respectively, and both mapped to chromosome 5 of the mungbean reference (Fig. 5). In the present study, 67 bruchid-resistance–associated genes, including DEGs and SCPs, were mapped to chromosome 5 of mungbean. The mapping results revealed a striking difference in promoter region of *g39185* between RIL59 and VC1973A. A similar phenomenon was observed with the promoters of *g34480* and the gene body of *g28730* (Fig. 6). These results imply that the genome structure at these positions differs between RIL59 and VC1973A, which might be related to the difference in resistance against bruchids.

### Generation of bruchid-resistance–associated markers

**Fig. 7:**
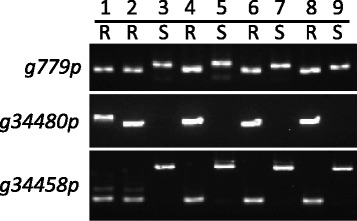
Bruchid-resistant–associated markers of mungbean. Markers designed from promoter sequences *g779p*, *g34480p*, and *g34458p* were used for selecting bruchid-resistant (R) and -susceptible (S) mungbean. The numbers 1 to 9 indicate different mungbean lines, named TC1966, RIL59, NM92, RIL38, RIL39, RIL54, RIL55, RIL153 and RIL156, respectively. PCR products of *g779p* and *g34458p* were analyzed on 4 % agarose gel and that of *g34480p* on 1 % agarose gel

From the bruchid-resistance–associated NVs, we selected long sequence indels and designed primers (Additional file 8: Table S8) for PCR-based molecular markers. Three markers derived from NVs on promoters of DEGs could distinguish R and S mungbean well between RIL59, two parents and three sets of RILs (Fig. 7). Marker *g779p* produced a smaller band in R than S mungbean. Marker *g34480p* produced a band only in R mungbean, as expected, but a smaller size in RILs than TC1966. Marker *g34458p* produced a small band in R mungbean and a large band in S mungbean. Further applying these markers together with the two bruchid-resistance–associated markers, the CAP marker OPW02a4 and SSR marker DMB-SSR158 [15, 17], to 61 RILs revealed DMB-SSR158 with the highest accuracy, 98.3 %, in selecting mungbean with bruchid resistance. The CAP marker OPW02a4, analyzed by digesting the PCR products with *Hae*III restriction enzyme, exhibited 73.7 % accuracy. The new developed markers *g779p* and *g34480p* exhibited 93.4 % accuracy, which was better than the 80.3 % accuracy of marker *g34458p* (Additional file 9: Table S9).

## Discussion

### Genome size of mungbean

The genome size, DNA quantity, or so-called C-value is important for genome polyploidy, phylogenetic and taxa research [30–32]. Recently, a reliable genome size for achieving the correct coverage and estimating the percentage of repeated sequences of a genome has become an important parameter for planning next-generation sequencing (NGS) experiments. Many methods have been used to estimate the genome size of organisms. Besides k-mer frequency distribution analysis together with NGS, flow cytometry has become the most popular method for estimating genome size [33] and is superior to other methods such as DNA phosphate content measurement [34], analysis of reassociation kinetics [35], pulsed-field gel electrophoresis [36] and image analysis of Feulgen photometry [37] because of its convenience, fast processing and reliability [38, 39].

The same flow cytometry system should be used for comparing plant genome sizes [40] and should avoid the use of an animal genome as a reference [33]. With these recommendations, the genome size estimation for the mungbean lines in our study varied by more than 60 Mb, from 493.6 to 554.7 Mb (Table 1), whereas previous reports estimated the genome sizes between 470 and 579 Mb [41, 42]. The large variation in estimations between the studies may be due to variation in mungbean lines and different strategies and methods used for analyses. The genome size of mungbean VC1973A was estimated at 579 Mb by flow cytometry with nuclei from chicken red blood cells used as an internal standard [28, 41], even though the use of animal genomes as a standard is not recommended for plant genome size prediction. The 25-base k-mer frequency distribution in NGS provided an estimated genome size of 548 Mb for V1973A [28], which is slightly larger than that by flow cytometry (Table 1). Similarly, the estimated genome size for the wild mungbean TC1966 was about 494 Mb in our flow-cytometry research but 501 Mb by 25-base k-mer frequency distribution [28]. In contrast, our k-mer frequency distribution provided a size estimate of 452 Mb for RIL59, smaller than by flow cytometry (Table 1). The purity of the constructed DNA libraries for NGS would affect the reliability of k-mer frequency distribution used to estimate genome size [30]. In our study, we used a flow-cytometry method with Arabidopsis nuclei as an internal standard to estimate the mungbean genome size and for a more reliable explanation of the NGS data obtained from RIL59.

Variation in the quantity of repetitive DNA sequences is the main factor determining genome size [43]. This fact was not true for the mungbean lines in our study. RIL59, with a larger genome than that for VC1973A or TC1966, contained only 40.45 % repetitive sequences as compared with 50.1 % and 46.9 % for VC1973A and TC1966, respectively. The difference in proportion of repetitive sequences in TC1966 and its offspring RIL59 suggests that heterozygosity of the genome in a hybrid can lead to loss of repetitive sequences.

### Genome assembly and gene annotation

Our genome assembly of RIL59 is comparable to that of VC1973A, which had 2748 scaffolds with N50 length of 1.52 Mb and 80 % genome coverage [28]. We made available a draft genome of a bruchid-resistant variety. Comparing this genome with the available sequence from bruchid-susceptible VC1973A can reveal genomic regions responsible for resistance. The annotated 36,939 genes in RIL59 are 14,512 genes greater than that reported for VC1973A [28]. The larger number of annotated genes could be due to the inclusion of more varied tissues and developmental stages of RIL59 than in the previous study, for a broader capture of different genes. We included RNA from seeds, different developmental stages of pods, 2- to 7-day-old seedlings and 1-month-old whole plants, for broader range of developmental stages and tissues and probably a more complete RNA population. The genome sequence information for the bruchid-resistant RIL59 and a more complete gene annotation of mungbean will contribute to improving “omic” research and promoting the breeding of mungbean in all aspects.

### DEGs and SCPs together maintain transcript diversity in bruchid-resistant mungbean

Research of bruchid resistance has focused on breeding and developing molecular markers [14–16]. Studies of resistance mechanisms at the transcriptomic and proteomic levels were attempted but did not reach final conclusions [21, 22]. We found that DEGs and SCPs might be strategies bruchid-resistant mungbean uses to retain transcript diversity and specificity. Therefore, further proteomic research of mungbean for bruchid resistance should consider both the effects of SCPs and quantity of differential proteins. The DEGs and SCPs related to bruchid resistance might be overestimated in our research for a few reasons. First, based on genetic and QTL studies [16–19], a major *Br* locus and two minor loci that might contain one to a few genes were proposed to be responsible for bruchid resistance in mungbean. Second, the RILs we used were not near-isogenic lines. Thus, not all of the identified DEGs and SCPs are likely to be directly associated with bruchid resistance. Third, nonsynonymous substitution due to NVs indeed may not affect the protein function and further biochemical characters in mungbean seeds. Further functional study of the selected DEGs and SCPs will help to evaluate their roles in bruchid resistance of mungbean.

Although the transcriptome of mature mungbean seeds most likely reflects what seeds prepared for the upcoming germination and may not be directly related to bruchid resistance of mungbean, the conserved expression pattern of DEGs implies that NVs on their promoters and those on SCPs can be potential molecular markers for mungbean breeding. Use of the promoter-based markers designed from bruchid-resistance–associated genes can indeed offer a high selection rate of bruchid-resistant mungbean.

Both DEGs and SCPs are involved in similar functional gene categories and conserved metabolic pathways, which implies intrinsic differences between bruchid-resistant and -susceptible mungbean. Whether these intrinsic differences of mungbean represent modifier factors or minor loci modulating bruchid resistance described in previous investigations [16, 17] needs further evaluation. However, almost one-quarter of the DEGs do not harbor NVs on their promoter regions, which suggests alternative mechanisms for differential regulation than sequence variation in these regions involved in regulating the expression of these genes.

### The search for a *Br* gene responsible for bruchid resistance of mungbean

Great efforts have been invested in mapping a *Br* gene in mungbean [19]. First, a single *Br* gene was proposed in TC1966, and later additional minor modifier factors were postulated [16, 17, 44]; nevertheless, the nature of the *Br* gene(s) of TC1966 remained unclear. Biochemically, several *Br* candidate genes highly associated with bruchid resistance have been reported, including the *Va* gene for vignatic acid biosynthesis [20]; vicilins, also known as 7S storage globulins [22]; and the cysteine-rich protein VrD1 [21]. Also, resistance-related proteins including chitinase, beta-1,3-glucanase and peroxidase were proposed to play a role in bruchid resistance of mungbean [22]. However, in cowpea and common bean, protease and amylase inhibitors, lectins, chitinases, and beta-1,3-glucanases are ineffective against *C. maculatus* and *Z. subfasciatus* [11]. In our research, only two genes that likely contain inhibitor activities were among the DEGs and a further two among SCPs. The two DEGs were downregulated in bruchid-resistant mungbean, so they might not be involved in resistance, whereas the role of the two detected SCPs encoding inhibitor-like proteins needs further study. None of the other previously proposed *Br* candidate genes was identified as a DEG or SCP, which reduces the probability that they play an important role in bruchid resistance of mungbean. Thus, the identity of a *Br* gene remains elusive.

From our research, the 91 DEGs and 148 SCPs are potential *Br* candidates. The mapping results narrowed the number of candidates to 67 on chromosome 5, where the two bruchid-resistant–associated markers, DMB-SSR158 and OPW02a4, are located. Hence, genes located near the markers are potential *Br* genes. However, the genome of mungbean is still incomplete, with many scaffold sequences that could not be assigned to the mungbean genome. Genes mapping to scaffolds cannot be eliminated from the putative *Br* gene list. Different mungbean accessions showed different genome sizes. Therefore, more genome sequencing is necessary to complete the genome assembly of mungbean, especially for TC1966 and other bruchid-resistant sources, for facilitating *Br* gene identification and mungbean breeding and improvement.

### TEs and bruchid resistance

Almost half of the genome of mungbean contained repetitive sequences in most TEs, including retrotransposons, transposons and miniature inverted-repeat TEs (MITEs). These elements are ubiquitous in eukaryotic genomes, although the content varies among the different organisms. They can represent 20 % of the genome, as for the *Drosophila melanogaster* genome [45], or 85 % for the *Zea mays* genome [46]. TEs are believed to be the major determinant of genome size [47].

Repetitive sequences, previously considered “junk DNA”, were found to function in modifying the genome structure and gene function and regulating gene expression [48]. The involvement of the non-LTR retrotransposon *CDT-1* in desiccation tolerance of *Craterostigma plantagineum* by mediating small RNA illustrated that TEs regulate plant stress resistance [49]. Could TEs also be involved in bruchid-resistance? We found that genes encoding TEs were DEGs and SCPs when comparing bruchid-resistant and -susceptible lines. In addition, the markers derived from TE sequences well distinguished bruchid-susceptible from –resistant mungbean lines. More studies are required to clarify how TEs are implicated in mungbean resistance or represent modifier factors for this trait.

## Conclusion

Here we provide whole-genome scaffold sequences for a bruchid-resistant mungbean line and increase the annotation of mungbean genes. We obtained a list of putative *Br* genes and candidates of molecular markers for selecting resistant lines and proposed that besides the *Br* gene, intrinsic differences caused by DEGs and SCPs of mungbean and TEs are most likely the modifier factors determining bruchid resistance. As expected, when comparing only a few selected lines with contrasting resistance phenotypes, the identified sequence variations spanned the whole chromosome. However, analysis of all DEG, SCP and NV data revealed factors located on chromosome 5 involved with resistance. More sequence information from different bruchid-resistant sources are needed to facilitate and promote mungbean research and crop improvement.

## Methods

### Plant materials and assessment of bruchid resistance

Mungbean (*Vigna radiata* [L.] R. Wilczek) of the bruchid-susceptible variety NM92, bruchid-resistant accession TC1966 (*V. radiata* var. *sublobata*), their 12-inbred-generation progeny (F_12_) RILs [15], the previously sequenced mungbean line VC1973A [28] and the two BRUCHID-resistant lines *V. radiata* V2802 and V2709 [4] were used as plant materials. All the plant materials used were from the support of AVRDC-The World Vegetable Center. Plants were grown and seeds without disease were harvested in greenhouses at AVRDC [17]. Assay for bruchid resistance with 40 seeds was performed in three replicates as described [15]. Seeds with 0 % damage were defined as resistant, with more than 80 % damage defined as susceptibility and damage between 0 % to 80 % defined as moderately resistant. DNA extracted from RIL59, TC1966 and NM92 was prepared for genome sequencing.

### Estimation of genome size by flow cytometry and k-mer distribution

Fresh tender leaves of TC1966, NM92, RIL59, VC1973A, V02802 and V02709 were harvested and the nuclear DNA content was estimated as described [33] with minor modifications. Fresh leaf sections (1.0 cm^2^) were chopped with use of a new razor blade in 1 mL ice-cold Tris-MgCl_2_ buffer (200 mM Tris, 4 mM MgCl-6H_2_O, 0.5 % triton X-100, pH: 7.5). After filtering through a 20-μm nylon mesh, the sample was stained with propidium iodide solution (50 μg/mL) containing RNase at 50 μg/mL. *Arabidopsis thaliana* (Columbia, 0.412 pg/2C) was used as a reference for estimating the mungbean genome size [38]. All samples were analyzed on a MoFlo XDP Cell Sorter (Beckman Coulter) coupled with a Quanta SC (Beckman Coulter) at the Flow Cytometry Analysis and Sorting Services, Institute of Plant and Microbial Biology (IPMB), Academia Sinica (AS), Taiwan. Additionally, the genome size of RIL59 after sequencing was estimated by analyzing the k-mer distribution by use of the KmerGenie program [50].

### DNA and RNA extraction

DNA was extracted from 0.5 g of the first leaves of 7-day-old plants of TC1966, NM92, RIL 59 and the three sets of RILs with use of the Plant Genomic DNA Extraction Minprep kit (Viogene, Taipei). RNA was extracted from 1-month-old whole plants, 2- to 7-day-old seedlings, open flowers and flower buds and pods at a series of developmental stages and from seeds of the bruchid-resistant line RIL59. RNA was also extracted from seed of bruchid-resistant and -susceptible parents (TC1966, NM92) and pairs of resistant and susceptible RILs, RIL38 and RIL39, RIL54 and RIL55, and RIL153 and RIL156. Each RIL pair originated from the same F2 plant and possessed the allele of the bruchid-resistant or -susceptible parent at any locus. Total RNA was extracted by use of the pine tree method with minor modifications [51]. DNA contamination of the RNA sample was removed with use of the TURBO DNA-free Kit (Ambion). RNA quality was analyzed on a Bioanalyzer RNA 6000 NanoChip (Agilent Technologies, Santa Clara, CA) coupled with an Agilent 2100 Bioanalyzer (Agilent Technologies) at the DNA Microarray Core Laboratory, IPMB, AS, Taiwan.

### Nucleotide sequencing and genome assembly

DNA and RNA sequencing involved an Illumina Hiseq2000 platform. Two paired-end and two mate-pair libraries were constructed from RIL59 DNA (Additional file 1: Table S1). The DNA was randomly fragmented and size-fractionated by electrophoresis. DNA fragments of 5 K, 2 K, 500 and 180 bp were purified and ligated with adapters to generate libraries for Hiseq sequencing. The DNA extracted from NM92 and TC1966 was used to construct a 500-bp library for Hiseq sequencing. The DNA sequence data were quality-filtered (25 of the first 35 bases at the 5′ end with a Phred quality score > 30 for read retention), and reads with ambiguous base-calls and > 85 % low complexity sequences were discarded. Contigs and scaffolds were assembled by use of ALLPATHS [52].

Two types of libraries were prepared for RNA sequencing (Additional file 1: Table S1). The total RNA extracted from 1-month-old whole plants, 2- to 7-day-old seedlings, flowers and pods at a series of developmental stages of RIL59 underwent rRNA removal, fragmentation, first- and second-strand cDNA synthesis, adapter ligation, PCR amplification and sequencing, and RNA from seeds of two parents and 10 RILs (Additional file 1: Table S1) was submitted to poly(A) RNA enrichment and fragmentation before sequencing.

### Gene annotation

Repeats were masked on the assembled genome by use of RepeatMasker [53] and RepeatModeler [54] with *de novo* repeat prediction along with the TIGR plant repeat database [55] and Repbase (2014/01/31). Quality-filtered RNA-seq reads of RIL59 were aligned to the repeat masked genome by use of STAR [56]. Transcript assembly for exon sequences involved use of Cufflinks [57]. NCBI soybean refseq protein sequences were aligned to the repeat masked genome by using exonerate [58]. The results of the alignments with the RNA sequences, the soybean refseq protein sequences and the assembled transcript sequences were used to generate extrinsic data for the gene prediction tool Augustus [59]. Transcripts from predicted gene-models were aligned against the NCBI non-redundant set of proteins by using Blastx (E-value 1e^−5^) to find homologues. The best alignment for each transcript was retained as an annotation. For functional annotation, Blast analysis of bruchid-resistant–associated genes involved use of Blastn for nucleotides and Blastx for protein homologs and putative function domains. The functional categories of a gene were determined by referring to the gene and protein description and the putative functional domain, if present, and were based on information from the KEGG website (http://www.genome.jp/kegg/). The best Blastx hit plus the functional description is presented as Blast results.

### RNA-seq analysis

The RNA-seq reads for all samples (Additional file 1: Table S1) were trimmed for low-quality bases and then aligned individually for each sample to the set of annotated transcripts by using BWA MEM [60]. For each dataset, transcript quantification involved use of eXpress [61] to calculate the transcripts per million (TPM) for each transcript. Fold change (FC) of gene expression was determined by calculating the ratio of TPM for resistant and susceptible samples. A transcript was denoted as up- or downregulated if the ratio was > 2 or < 0.5, respectively; otherwise, it was denoted as non-differentially expressed.

We also used DESeq for differential expression analysis of transcripts by calculating the total read counts of a gene in each sample. The DESeq analysis used the default parameter described previously [29]. A transcript was denoted as a DEG with P_adj_ < 0.1, *P* < 0.05 [29], with FC resistance to susceptible > 2 or < 0.5, respectively. Otherwise, a transcript was denoted as non-differentially expressed.

### RT-qPCR validation and genomic PCR for genotyping and sequencing

RNA samples extracted from seeds of RIL59, TC1966, NM92 and 3 sets of RILs were used as templates for RT-pPCR analysis. Primer pairs (Additional file 8: Table S8) for each gene were designed with use of Primer Express v2.0 (Applied Biosystems). qPCR involved use of the QuantStudio 12 K Flex System (Applied Biosystems) under a cycling profile of 50 °C for 2 min, then 95 °C for 10 min, and 40 cycles of 95 °C for 15 s and 60 °C for 1 min. The relative quantity (RQ) of gene expression was determined by the 2^−ΔΔCT^ method (ΔCT = CT of interest gene − CT of control gene; ΔΔCT = ΔCT of experimental mungbean − ΔCT of control mungbean) [62]. Some genes were not detected in all mungbean lines, so we denoted their CT value as 40 to easily compare the RQ of gene expression between different mungbean lines. The expression of the mungbean acting gene, *g12676*, was used as an internal control to normalize the expression of genes tested. Three experimental repeats were performed.

Genomic PCR was performed with primer pairs (Additional file 8: Table S8) designed for molecular marker validation and for DNA sequencing. For marker testing, the PCR products for the CAP marker OPW02a4 were further digested with the *Hae*III restriction enzyme (New England) at 37 °C following the user instructions. The markers were analyzed on an agarose gel or a 6 % polyacrylamide gel. For sequence comparison, the PCR products were sequenced and the obtained DNA sequences were aligned with use of ContigExpress in Vector NTI Suite 9 (Invitrogen) to indicate NVs between R and S mungbean.

### Mapping of bruchid-resistance–associated genes on mungbean pseudochromosome

The BLAT program [63] was used for gene location mapping. The coding sequences and promoter region (2-kb upstream if any) of genes were aligned against the draft mungbean VC1973A genome [28]. The applied tile size was 11 and the step size was set to 5. The pslReps option was used to choose the possible location with use of the singleHit option.

## Ethics approval and consent to participate

Not applicable.

## Consent for publication

Not applicable.

## Availability of data and materials

This Whole Genome Shotgun project of the genome assembly for bruchid-resistant mungbean RIL59 has been deposited at DDBJ/EMBL/GenBank under the accession LJIH00000000. The RNA-seq data for mungbean lines are available form the NCBI Bioproject: PRJNA276314.

## Additional files

Additional file 1: Table S1. Sequencing libraries for RIL59 genome assembly and transcriptome analysis. (DOC 42 kb) Additional file 2: Table S2. Differentially expressed genes (DEGs) associated with bruchid resistance of mungbean. (XLS 67 kb) Additional file 3: Table S3. DEGs involved in metabolic pathways. (XLS 31 kb) Additional file 4: Table S4. Nucleotide variations (NVs) in the 2-kb promoter region of DEGs. (XLS 79 kb) Additional file 5: Table S5. Transcriptome-based NVs on exons of genes between bruchid-susceptible mungbean NM92 and bruchid-resistant mungbean TC1966 and RIL59. (XLS 57 kb) Additional file 6: Table S6. Bruchid-resistance–associated sequence-changed-protein genes (SCPs) of mungbean. (XLS 82 kb) Additional file 7: Table S7. SCPs involved in metabolic pathways. (XLS 35 kb) Additional file 8: Table S8. Primers used in the experiments. (DOCX 89 kb) Additional file 9: Table 9. Marker test of *g779p*, *g34480p*, *g34458p*, DMB-SSR158 and OPW02a4 on mungbean recombinant inbred lines (RILs). (XLSX 432 kb)

## Abbreviations

*Br*: bruchid-resistant gene
CAP: cleaved amplified polymorphic DNA
DEG: differential expressed gene
FC: fold change
Gb: giga base pairs
kb: kilo base pairs
LTR: long tandem repeat
Mb: mega base pairs
MITE: miniature inverted-repeat transposable elements
NGS: next generation sequencing
NV: nucleotide variation
R: bruchid-resistant
RIL: recombinant inbred line
S: bruchid-susceptible
SCP: sequence-changed-protein gene
SSR: simple sequence repeat
TE: transposable element
TPM: transcripts per million

## References

[1] Bains K, Yang RY, Shanmugasundaram S. High iron mungbean recipes for north India. AVRDC-The World Vegetable Center AVRDC publication, Tainan. 2003;03–562.34:3.

[2] Weinberger K (2005). Assessment of the nutritional impact of agricultural research: the case of mungbean in Pakistan. Food Nutr Bull.

[3] Tang D, Dong Y, Ren H, Li L, He C (2014). A review of phytochemistry, metabolite changes, and medicinal uses of the common food mung bean and its sprouts (*Vigna radiata*). Chem Cent J.

[4] Somta P, Ammaranan C, Ooi PAC, Srinives P (2007). Inheritance of seed resistance to bruchids in cultivated mungbean (*Vigna radiata*, L. Wilczek). Euphytica.

[5] Lambrides CJ, Godwin ID, Kole C (2007). Mungbean. Genome mapping and molecular breeding in plants. Pulses, sugar and tuber crops.

[6] Fernandez GCJ, Talekar NS, Fujii K, Gatehouse AMR, Johnson CD, Mitchell R, Yoshida T (1990). Genetics and breeding for bruchid resistance in Asiatic *Vigna* species. Bruchids and legumes: economics, ecology and coevolution.

[7] Southgate BJ, Singh SR, van Emden HF, Taylor TA (1978). The importance of the bruchidae as pests of grain legumes, their distribution and control. Pests of Grain Legumes: Ecology and Control.

[8] Hong MG, Kim KH, Ku JH, Jeong JK, Seo MJ, Park CH (2015). Inheritance and quantitative trait loci analysis of resistance genes to bruchid and bean bug in mungbean (*Vigna radiata* L. Wilczek). Plant Breed Biotechnol.

[9] Daglish GJ, Erbacher JM, Eelkema M (1993). Efficacy of protectants against *Callosobruchus Phaseoli* (Gyll) and *C. Maculatus* (F) (Coleoptera, Bruchidae) in Mungbeans. J Stored Prod Res.

[10] Gbaye OA, Millard JC, Holloway GJ (2011). Legume type and temperature effects on the toxicity of insecticide to the genus *Callosobruchus* (Coleoptera: Bruchidae). J Stored Prod Res.

[11] Sales MP, Gerhardt IR, Grossi-de-Sa MF, Xavier J (2000). Do legume storage proteins play a role in defending seeds against bruchids?. Plant Physiol.

[12] Soundararajan RP, Chitra N, Geetha S, Poorani J (2012). Biological control of bruchid *Callosobruchus maculates* (F.) in blackgram. J Biopest.

[13] Tomooka N, Lairungreang C, Nakeeraks P, Egawa Y, Thavarasook C (1992). Development of bruchid-resistant mungbean line using wild mungbean germplasm in Thailand. Plant Breed.

[14] Fujii K, Ishimoto M, Kitamura K (1989). Patterns of resistance to bean weevils (Bruchidae) in *Vigna*-*radiata*-*mungo*-*sublobata* complex inform the breeding of new resistant varieties. Appl Entomol Zool.

[15] Chen HM, Liu CA, Kuo CG, Chien CM, Sun HC, Huang CC (2007). Development of a molecular marker for a bruchid (*Callosobruchus chinensis* L.) resistance gene in mungbean. Euphytica.

[16] Kitamura K, Ishimoto M, Sawa M (1988). Inheritance of resistance to infestation with azuki bean weevil in *Vigna*-*sublobata* and successful incorporation to *Vigna*-*radiata*. Jpn J Breed.

[17] Chen HM, Ku HM, Schafleitner R, Bains TS, Kuo CG, Liu CA (2013). The major quantitative trait locus for mungbean yellow mosaic Indian virus resistance is tightly linked in repulsion phase to the major bruchid resistance locus in a cross between mungbean [*Vigna radiata* (L.) Wilczek] and its wild relative *Vigna radiata* ssp. *sublobata*. Euphytica.

[18] Ishimoto M, Kitamura K (1993). Inhibitory Effects of adzuki bean weevil-resistant mungbean seeds on growth of the bean bug. Jpn J Breed.

[19] Young ND, Kumar L, Menanciohautea D, Danesh D, Talekar NS, Shanmugasundarum S (1992). RFLP mapping of a major bruchid resistance gene in mungbean (*Vigna*-*Radiata*, L-Wilczek). Theor Appl Genet.

[20] Kaga A, Ishimoto M (1998). Genetic localization of a bruchid resistance gene and its relationship to insecticidal cyclopeptide alkaloids, the vignatic acids, in mungbean (*Vigna radiata* L. Wilczek). Mol Gen Genet.

[21] Chen KC, Lin CY, Kuan CC, Sung HY, Chen CS (2002). A novel defensin encoded by a mungbean cDNA exhibits insecticidal activity against bruchid. J Agr Food Chem.

[22] Khan MMK, Khan A, Ishimoto M, Kitamura K, Komatsu S (2003). Proteome analysis of the relationship between bruchid-resistant and -susceptible mungbean genotypes. Plant Genetic Resources.

[23] Ishimoto M, Sato T, Chrispeels MJ, Kitamura K (1996). Bruchid resistance of transgenic azuki bean expressing seed alpha-amylase inhibitor of common bean. Entomol Exp Appl.

[24] Shade RE, Schroeder HE, Pueyo JJ, Tabe LM, Murdock LL, Higgins TJV (1994). Transgenic pea-seeds expressing the alpha-amylase inhibitor of the common bean are resistant to bruchid beetles. Bio-Technol.

[25] Piergiovanni AR, Dellagatta C, Sergio L, Perrino P (1994). High antinutrient levels and bruchid resistance of cowpea (*Vigna*-*Unguiculata*) seeds. Euphytica.

[26] Fernandes KVS, Sabelli PA, Barratt DHP, Richardson M, Xavierfilho J, Shewry PR (1993). The resistance of cowpea seeds to bruchid beetles is not related to levels of cysteine proteinase-inhibitors. Plant Mol Biol.

[27] Miura K, Ishimoto M, Yamanaka N, Miyazaki S, Hiramatsu M, Nakajima Y (1996). Effects of bruchid-resistant mungbean meal on growth and blood-biochemical values in mice. JIRCAS Journal.

[28] Kang YJ, Kim SK, Kim MY, Lestari P, Kim KH, Ha BK, et al. Genome sequence of mungbean and insights into evolution within *Vigna* species. Nat Commun. 2014; doi:10.1038/ncomms6443 10.1038/ncomms6443 PMC4241982 25384727

[29] Anders S, Huber W. Differential expression analysis for sequence count data. Genome Biol. 2010;11(10):R106. 10.1186/gb-2010-11-10-r106 PMC3218662 20979621

[30] Guo LT, Wang SL, Wu QJ, Zhou XG, Xie W, Zhang YJ (2015). Flow cytometry and K-mer analysis estimates of the genome sizes of *Bemisia tabaci* B and Q (Hemiptera: Aleyrodidae). Front Physiol.

[31] Barow M, Meister A (2003). Endopolyploidy in seed plants is differently correlated to systematics, organ, life strategy and genome size. Plant Cell Environ.

[32] Kondorosi E, Roudier F, Gendreau E (2000). Plant cell-size control: growing by ploidy?. Curr Opin Plant Biol.

[33] Dolezel J, Greilhuber J, Suda J (2007). Estimation of nuclear DNA content in plants using flow cytometry. Nat Protoc.

[34] Shapiro HS, Sober HA (1970). Nucleic acids. CRC handbook of biochemistry. selected data for molecular biology.

[35] Leutwiler LS, Houghevans BR, Meyerowitz EM (1984). The DNA of *Arabidopsis*-*thaliana*. Molecular & General Genetics.

[36] Rydkina E, Roux V, Raoult D (1999). Determination of the genome size of *Ehrlichia* spp., using pulsed field gel electrophoresis. Fems Microbiol Lett.

[37] Voglmayr H, Greilhuber J (1998). Genome size determination in Peronosporales (Oomycota) by Feulgen image analysis. Fungal Genet Biol.

[38] Schmuths H, Meister A, Horres R, Bachmann K (2004). Genome size variation among accessions of *Arabidopsis thaliana*. Ann Bot-London.

[39] Wilhelm J, Pingoud A, Hahn M (2003). Real-time PCR-based method for the estimation of genome sizes. Nucleic Acids Res.

[40] Dolezel J, Greilhuber J, Lucretti S, Meister A, Lysak MA, Nardi L (1998). Plant genome size estimation by flow cytometry: Inter-laboratory comparison. Ann Bot-London.

[41] Arumuganathan K, Earle E (1991). Nuclear DNA content of some important plant species. Plant Mol Biol Rep.

[42] Murray MG, Palmer JD, Cuellar RE, Thompson WF (1979). Deoxyribonucleic acid sequence organization in the mung bean genome. Biochemistry.

[43] Flavell R (1980). The molecular characterization and organization of plant chromosomal DNA-sequences. Annu Rev Plant Phys..

[44] Sarkar S, Ghosh S, Chatterjee M, Das P, Lahari T, Maji A (2011). Molecular markers linked with bruchid resistance in *Vigna radiata* var. *Sublobata* and their validation. J Plant Biochem Biot.

[45] Bergman CM, Quesneville H, Anxolabehere D, Ashburner M (2006). Recurrent insertion and duplication generate networks of transposable element sequences in the *Drosophila melanogaster* genome. Genome Biol.

[46] Schnable PS, Ware D, Fulton RS, Stein JC, Wei FS, Pasternak S (2009). The B73 maize genome: Complexity, diversity, and dynamics. Science.

[47] Piegu B, Guyot R, Picault N, Roulin A, Saniyal A, Kim H (2006). Doubling genome size without polyploidization: Dynamics of retrotransposition-driven genomic expansions in Oryza australiensis, a wild relative of rice. Genome Res.

[48] Bennetzen JL (2000). Transposable element contributions to plant gene and genome evolution. Plant Mol Biol.

[49] Furini A, Grandbastien MA, Casacuberta JM (2012). Retrotransposons and the eternal leaves. Plant transposable elements.

[50] Chikhi R, Medvedev P (2014). Informed and automated k-mer size selection for genome assembly. Bioinformatics.

[51] Chang S, Puryear J, Cairney J (1993). A simple and efficient method for isolating RNA from pine trees. Plant Mol Biol Rep.

[52] Butler J, MacCallum I, Kleber M, Shlyakhter IA, Belmonte MK, Lander ES (2008). ALLPATHS: de novo assembly of whole-genome shotgun microreads. Genome Res.

[53] Smit AFA, Hubley R, Green P. Repeatmasker open-3.0. http://www.repeatmasker.org. 1996–2010. Accessed 31 Jan 2014.

[54] Smit AFA, Hubley R. Repeatmodeler open-1.0. http://www.repeatmasker.org. 2008–2010. Accessed 31 Jan 2014.

[55] Ouyang S, Buell CR (2004). The TIGR plant repeat databases: a collective resource for the identification of repetitive sequences in plants. Nucleic Acids Res.

[56] Dobin A, Davis CA, Schlesinger F, Drenkow J, Zaleski C, Jha S (2013). STAR: ultrafast universal RNA-seq aligner. Bioinformatics.

[57] Trapnell C, Roberts A, Goff L, Pertea G, Kim D, Kelley DR (2012). Differential gene and transcript expression analysis of RNA-seq experiments with TopHat and Cufflinks. Nat Protoc.

[58] Slater GS, Birney E (2005). Automated generation of heuristics for biological sequence comparison. BMC bioinformatics.

[59] Stanke M, Diekhans M, Baertsch R, Haussler D (2008). Using native and syntenically mapped cDNA alignments to improve de novo gene finding. Bioinformatics.

[60] Li H, Durbin R (2009). Fast and accurate short read alignment with Burrows-Wheeler transform. Bioinformatics.

[61] Roberts A, Pachter L (2013). Streaming fragment assignment for real-time analysis of sequencing experiments. Nat Methods.

[62] Livak KJ, Schmittgen TD (2001). Analysis of relative gene expression data using real-time quantitative PCR and the 2(−Delta Delta C(T)) method. Methods.

[63] Kent WJ (2002). BLAT - The BLAST-like alignment tool. Genome Res.

