# Supplementary material for: Genomic and transcriptomic comparison of nucleotide variations for insights into bruchid resistance of mungbean (Vigna radiata [L.] R. Wilczek)
Source: BMC Plant Biol. 2016 Feb 17;16:46. doi: 10.1186/s12870-016-0736-1 (PMC4756517; doi:10.1186/s12870-016-0736-1)
Supplement: Additional file 1: Table S1. — Sequencing libraries for RIL59 genome assembly and transcriptome analysis. (DOC 42 kb) [file 12870_2016_736_MOESM1_ESM.doc]

Table S1. Sequencing libraries for RIL59 genome assembly and transcriptome analysis.

|  | **Library** | **Type** | **Read Length** | **No. Read Pairs** | **Purpose** |
| --- | --- | --- | --- | --- | --- |
| DNA | 180 bp of RIL59 | paired-end | 100 bp | 150,208,142 | Genome assembly, genome comparison |
|  | 500 bp of RIL59 | paired-end | 100 bp | 142,122,577 | Genome assembly, genome comparison |
|  | 2k bp of RIL59 | mate-pair | 100 bp | 85,303,871 | Genome assembly, genome comparison |
|  | 5k bp of RIL59 | mate-pair | 100 bp | 72,861,175 | Genome assembly, genome comparison |
|  | 500 bp of TC1966 | paired-end | 100 bp | 34,167,593 | Genome comparison |
|  | 500 bp of NM92 | paired-end | 100 bp | 37,959,045 | Genome comparison |
| RNA | RIL59 pod | paired-end | 100 bp | 77,462,222 | Gene annotation |
|  | RIL59 flower | paired-end | 100 bp | 80,643,135 | Gene annotation |
|  | RIL59 2-7 days seedlings | paired-end | 100 bp | 81,163,149 | Gene annotation |
|  | RIL59 1 month plant | paired-end | 100 bp | 86,826,408 | Gene annotation |
|  | NM92 seeds | paired-end | 151 bp | 30,234,891 | Gene annotation, transcriptomic comparison |
|  | TC1966 seeds | paired-end | 151 bp | 24,695,625 | Gene annotation, transcriptomic comparison |
|  | RIL59 seeds | paired-end | 151 bp | 29,369,177 | Gene annotation, transcriptomic comparison |
|  | RIL38 seeds | paired-end | 151 bp | 29,356,387 | Gene annotation, transcriptomic comparison |
|  | RIL39 seeds | paired-end | 151 bp | 28,011,113 | Gene annotation, transcriptomic comparison |
|  | RIL40 seeds | paired-end | 151 bp | 28,419,321 | Gene annotation |
|  | RIL54 seeds | paired-end | 151 bp | 31,866,190 | Gene annotation, transcriptomic comparison |
|  | RIL55 seeds | paired-end | 151 bp | 29,637,610 | Gene annotation, transcriptomic comparison |
|  | RIL56 seeds | paired-end | 151 bp | 29,909,434 | Gene annotation |
|  | RIL153 seeds | paired-end | 151 bp | 29,883,047 | Gene annotation, transcriptomic comparison |
|  | RIL155 seeds | paired-end | 151 bp | 26,152,681 | Gene annotation |
|  | RIL156 seeds | paired-end | 151 bp | 28,465,505 | Gene annotation, transcriptomic comparison |
