# Supplementary material for: Genomic and transcriptomic comparison of nucleotide variations for insights into bruchid resistance of mungbean (Vigna radiata [L.] R. Wilczek)
Source: BMC Plant Biol. 2016 Feb 17;16:46. doi: 10.1186/s12870-016-0736-1 (PMC4756517; doi:10.1186/s12870-016-0736-1)
Supplement: Additional file 8: Table S8. — Primers used in the experiments. (DOCX 89 kb) [file 12870_2016_736_MOESM8_ESM.docx]

**Table S8. Primers used in the experiments.**

| Primer | Sequence (5’ ~ 3’) | Purpose |
| --- | --- | --- |
| g636_C_F1 | gCT CgC AAA CAg AgA gAC CC | Genomic PCR |
| g636_C_R1 | gAg AAA CTA CTA CCA Cgg CC | Genomic PCR |
| g656_C_F1 | CCC AAT gCT CCT AAA ggT TgC | Genomic PCR |
| g656_C_R1 | gAA CAC AAA ATC TCT TCC CAg TC | Genomic PCR |
| g662_C_F1 | CCA ATg CgA TTC gCC ACC TC | Genomic PCR |
| g662_C_R1 | CCA AAA gAT CTg gCA AAg CAA AC | Genomic PCR |
| g662_C_F2 | TTg ATC ACT CAg ATT TCT TCT gTg | Genomic PCR |
| g662_C_R2 | CAT TCT TTT gCT TAC ATA Agg CTg | Genomic PCR |
| qg39185_F1 | GGG ATC CAC ACC ATG TTG C | RT-qPCR |
| qg39185_R1 | Tgg TCC CAg gCT TCA CTT Tg | RT-qPCR |
| qg779_F1 | CCA CCg TCA gTT CAC AgA gAA g | RT-qPCR |
| qg779_R1 | TCC CCA ATC ACA TCC TCC Tg | RT-qPCR |
| qg34458_F4 | TCA gCT TCT CAg CAT TAA CCA CTT | RT-qPCR |
| qg34458_R4 | TgC AAg gCA CTg AAA CTC CA | RT-qPCR |
| qg9801_F1 | ATg gAT TTg CTA AAA ggC gTT gT | RT-qPCR |
| qg9801_R1 | TCT CAT CCC gAA ggg CAg TA | RT-qPCR |
| qg17262_F1 | CgC AAA AAC AAC gAA gAA ggA | RT-qPCR |
| qg17262_R1 | ATC ACC CCT gTT ggA ATC AAT T | RT-qPCR |
| qg4739_F3 | CAA gAA Agg CCA TCA ACA TAC AAg T | RT-qPCR |
| qg4739_R3 | CgC TTC CTT gAT CAg ATA CCA TAA T | RT-qPCR |
| qg4706_F2 | Agg gCg AgC TAT gCg ATT C | RT-qPCR |
| qg4706_R2 | TCg gAg TCA CAg CCA CAg AA | RT-qPCR |
| qg34480_F1 | gAT TgC CTT gCA gAA TTA AgC A | RT-qPCR |
| qg34480_R1 | ggg TgC CTT TgT TTC AgC Ag | RT-qPCR |
| qg42613_F1 | TgC AgC AAA TCC TgT Tgg Ag | RT-qPCR |
| qg42613_R1 | CTC TCT gTg AAC CAg CAC CgT | RT-qPCR |
| qg19905_F1 | TTg AAA Tgg AAC CCC TCT ACA Tg | RT-qPCR |
| qg19905_R1 | CAg ATC CAA AAC AAg gCA gCT C | RT-qPCR |
| qg4736_F1 | AAA gTT ggg ATC ggT ggA ATC | RT-qPCR |
| qg4736_R1 | AAT TCC TCT TTC ACC CTC TgC A | RT-qPCR |
| qg4663_F1 | CTg ACT ATg ATg CTA TTg gTg gCT | RT-qPCR |
| qg4663_R1 | TgC gTC CTT TgA Tgg CAg A | RT-qPCR |
| qg40048_F3 | ACA gCg TgT Cgg CAg gAA | RT-qPCR |
| qg40048_R3 | CCA CgT TCA TCg CCT TTC TC | RT-qPCR |
| qg28764_F1 | TCT TTT TTA TTg TCT CTg gCC AAg A | RT-qPCR |
| qg28764_R1 | TTC ATC CCC gTg CAT gC | RT-qPCR |
| qg9806_F1 | ATg TTg CTC TAC CCT CAg TAT ggC | RT-qPCR |
| qg9806_R1 | ggC AAT ggg TTg gAA AAT gAT | RT-qPCR |
| qg728_F1 | AgC TgT CgC CAA CCA ACC T | RT-qPCR |
| qg728_R1 | TCA gAT CCC CCA gCA TCA AT | RT-qPCR |
| qg759_F1 | AgA Agg TgA TTg TCT gTT gCC A | RT-qPCR |
| qg759_R1 | AAT TTg CCg CAC CAg TAT CC | RT-qPCR |
| qg12676_F1 | TTC CTT TTC TTC gTT TTT CAT gCT | RT-qPCR |
| qg12676_R1 | AAA CCg Agg CAC CTA ATT CTT g | RT-qPCR |
| 779_pF1 | CTA ATA AAT CAT CTA TAC gTC TCT C | Molecular marker |
| 779_pR1 | ATT gCT ATT TAg CgA ATA ATA gTA C | Molecular marker |
| Vr34480_pF | AAT TCT TgA TTg gTC CAC ATg | Molecular marker |
| Vr34480_pR | AAA AAA TTA CAC CTC gTT Cg | Molecular marker |
| 34458_pF | ATg gTg TCA AAA gCA TTT TAC CAg | Molecular marker |
| 34458_pR | TAA CAA CTg gAA CTA CCT CTC g | Molecular marker |
| DMB-SSR158-F | Tgg AAA ATT TgC AgC AgT Tg | Molecular marker |
| DMB-SSR158-R | ATT gAT ggA ggg Cgg AAg TA | Molecular marker |
| OPW02a4-F | CCA AAg gAg TCg AgT gAA ACT | Molecular marker |
| OPW02a4-R | CAA CAA CCC TTC CTC TAT CTC | Molecular marker |
